# A Rare Case of Central Pontine Myelinolysis in Overcorrection of Hyponatremia with Total Parenteral Nutrition in Pregnancy

**DOI:** 10.1155/2015/940807

**Published:** 2015-12-22

**Authors:** Kalyana C. Janga, Tazleem Khan, Ciril Khorolsky, Sheldon Greenberg, Priscilla Persaud

**Affiliations:** Maimonides Medical Center, 4802 10th Avenue, Brooklyn, NY 11219, USA

## Abstract

A 42-year-old high risk pregnant female presented with hyponatremia from multiple causes and was treated with total parenteral nutrition. She developed acute hypernatremia due to the stage of pregnancy and other comorbidities. All the mechanisms of hyponatremia and hypernatremia were summarized here in our case report. This case has picture (graph) representation of parameters that led to changes in serum sodium and radiological findings of central pontine myelinolysis on MRI. In conclusion we present a complicated case serum sodium changes during pregnancy and pathophysiological effects on serum sodium changes during pregnancy.

## 1. Introduction

Hyponatremia is defined as a sodium concentration less than 135 mEq/L. If hyponatremia is not corrected appropriately, it can lead to significant clinical symptoms. During pregnancy, hyponatremia can be caused by various mechanisms. A reset osmostat phenomenon is one of the physiologic changes that occur during pregnancy. The osmotic threshold is decreased to a lower steady state value due to excess ADH release and a heightened thirst stimulus. This results in a decrease in the average plasma-osmolality by 5–10 mmol and the sodium concentration by up to 5 mmol/L [1].

Another cause of hyponatremia that is related to pregnancy is HG. HG is a complication of pregnancy that is associated with assisted reproduction techniques and multiple gestations [2, 3]. It is characterized by intractable vomiting often requiring parenteral nutrition and can have a profound effect on the patient's fluid and electrolyte status. It can result in malnutrition, hyponatremia, and low serum urea levels [4, 5]. Hyponatremia in HG is due to both hypovolemic stimulus of AVP and also low osmoles intake that impairs free water excretion. TPN is a form of nutritional support that contains lipid emulsions, amino acids, utilizable nitrogen, and nonprotein calorie sources such as glucose, electrolytes, and minerals. It is indicated when enteral nutrition is not possible [6]. It may be used in pregnancy complicated by severe HG when conservative treatment has failed [2]. Use of TPN can reverse weight loss and meet the specific requirements necessary for maternal anabolism and fetal embryologic growth [7]. Sodium may be increased in the TPN prescription. However, parenteral sodium should be administered carefully so as to avoid rapid and/or excessive correction to minimize the risk of CPM [8].

On the other end of the spectrum, dysregulation of the vasopressin system can lead to hypernatremia, defined as a sodium concentration above 145 mEq/L. Sepsis has been associated with decreased levels of vasopressin [9]. Proposed mechanisms include reduced production of vasopressin, depletion of vasopressin stores, inhibition of vasopressin release (in a septic state, the inhibition of vasopressin overcomes the excess vasopressin release in pregnancy), impaired baroreflex mediated vasopressin secretion, and increased vasopressin degradation [9]. Significant clinical consequences can occur due to a rapid increase in sodium levels. CPM is a rare syndrome that typically occurs as a consequence of rapid correction of hyponatremia. In a hyponatremic state of greater than 2-3-day duration, there is loss of osmotically active substances (sodium, potassium, chloride, and organic osmolytes such as myoinositol, glutamate, and glutamine), which normally protect against cerebral edema. If there is a rapid correction of hyponatremia, these substances (with the exception of sodium) are not replaced efficiently, leading to cerebral edema and CPM [10]. Hypertonic saline was not used to correct the sodium as it is only indicated if neurological symptoms prevail with the severe hyponatremia [6]. Although vasopressin receptor 2 antagonists such as tolvaptan and hypertonic saline are treatments for hyponatremia, they could not be used in this case. Tolvaptan is category C in pregnancy and can be used with caution but was contraindicated in this patient due to hypovolemia and excessive vomiting.

## 2. Case Description

A 42-year-old woman, G2P0 at 14 weeks of gestation with diamniotic-dichorionic twins from an in vitro fertilization, presented to the hospital with refractory hyperemesis gravidarum (HG). She had protein calorie malnutrition with a 25% decreased PO intake and 7% weight loss. The serum sodium concentration on admission was 125 mEq/L. During the hospital course, she was treated with normal saline and increased protein intake. Urine electrolytes and urine osmolality were measured showing urine sodium of 90 mmol/L, urine potassium of 11.5 mmol/L, and urine osmolality of 440 mOsm/kg. The patient's symptoms failed to improve and she had a peripherally inserted central catheter (PICC) placed and TPN was initiated. The patient experienced improvement in symptoms and normalization of the sodium in pregnancy, where the sodium corrected 0.3 mEq/hr during the first 24 hours. The sodium corrected by day 2 of this admission and persisted to be normal by day 5. The discharge was delayed due to home care issues.

**Figure 1:**
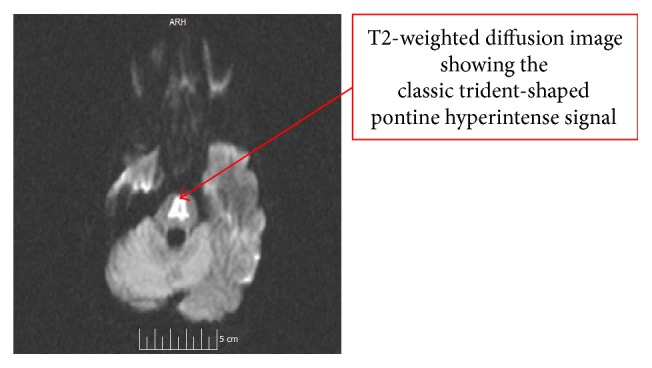
Initial MRI of brain.

**Figure 2:**
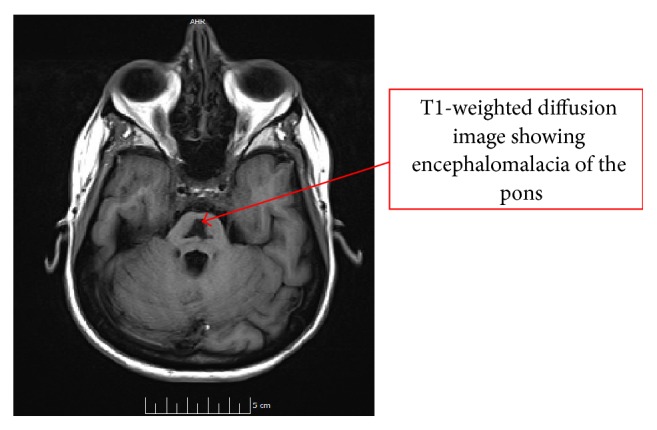
Repeat MRI of brain after recovery.

Two weeks later, the patient was readmitted for persistent HG, psychomotor retardation, intention tremor, fluctuating altered mental status, and incontinence of 2-day duration. On physical exam, the patient was tachycardic with stable blood pressure. Neurological exam demonstrated ankle clonus, hyperreflexia, saccadic breakdown of smooth pursuit, facial diparesis, and dysarthria. Laboratory data revealed leucocytosis of 16.8 and sodium of 163 mEq/L. She was found to have an infected PICC line with bacteremia and fungemia and met criteria for sepsis. An MRI of the brain was performed which revealed changes consistent with CPM (Figure 1). The patient's neurological symptoms persisted for 6 days into this admission and improved slowly as the sodium corrected from 163 mEq/L to 141 mEq/L. During this time, the patient also had hypokalemia to normokalemia ranging from 2.9 to 3.9 mmol/L. The potassium was supplemented with oral replacements as well as TPN which was constituted with 2.8–3.8 mEq/dL of potassium and other electrolytes such as calcium, magnesium, and phosphorus. During the hospitalization, urine osmolality was measured showing a level of 104 mOsm/kg. Urine output however could not be measured due to strict intensive care policies on minimizing Foley catheter placements. Subsequently, the patient underwent termination of pregnancy with improvement of CPM signs on MRI done 2 months later (Figure 2).

## 3. Discussion

In this case, hyponatremia that was caused by hypovolemia and low osmotic intake (e.g., proteins) in the setting of HG was treated with hydration, protein supplementation, and nausea control. Concentrated TPN, consisting of high salt and protein supplements, via PICC line was the treatment of choice. As the patient's serum sodium increased, the TPN prescription was recalculated with lower volume and a decreased salt load.

Although not in septic shock this patient had tachycardia, leucocytosis, and bacteremia with* Enterococcus faecalis* and fungemia with* Candida albicans* meeting the criteria for sepsis. Sepsis has been established as a cause of diabetes insipidus and could have led to the overcorrection of sodium leading to CPM (Figure 1). The proposed mechanisms are increased metabolic clearance rate via increased vasopressinase activity [11], decreased production, or decreased release of vasopressin from the pituitary gland.

In our patient, despite having several risk factors for developing CPM including protein denutrition and potassium depletion, the primary cause favors the rapid correction of hypovolemia. In addition, the contribution of high vasopressinase activity (and thus high vasopressin clearance) due to diamniotic-dichorionic gestation can also explain the acute hyponatremia [12]. This is supported by the decrease in urine osmolality from 440 mOsm/kg on the first admission to 104 mOsm/kg on the second admission.

**Figure 3:**
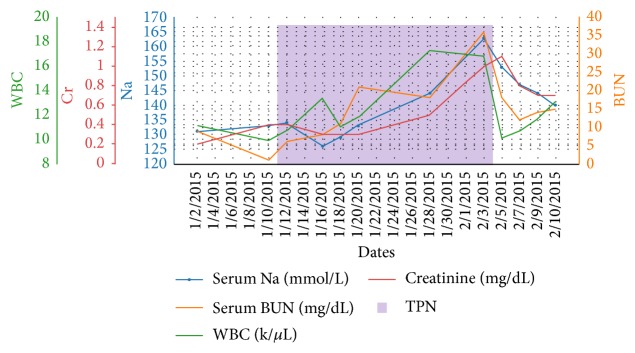
Correlation of serum Na, BUN, WBC, Cr, and TPN.

This patient's sodium was slowly corrected with hypotonic solution administration. Improvement in her neurological symptoms paralleled the correction of hypernatremia. A repeat MRI two months later showed resolution of CPM signs (Figure 2). Correlation of serum Na, BUN, WBC, Cr, and TPN is shown in Figure 3.

## 4. Conclusion

This case report describes the occurrence of rapid overcorrection of hyponatremia in pregnancy and sepsis. The outcome of treating hyponatremia in pregnancy associated with sepsis can be unpredictable. There is a high risk of overcorrection and developing hypernatremia given the complexities of the physiology of vasopressinase associated with pregnancy and sepsis. These risk factors should be taken into account when planning the rate of correction of sodium and volume in HG. In addition, serum sodium must be followed closely when correcting hyponatremia in this group of patients.
